# Plasmid-mediated colistin resistance and ESBL production in *Escherichia coli* from clinically healthy and sick pigs

**DOI:** 10.1038/s41598-022-06415-0

**Published:** 2022-02-14

**Authors:** Suthathip Trongjit, Pornchalit Assavacheep, Sukuma Samngamnim, Tran Hoang My, Vo Thi Tra An, Shabbir Simjee, Rungtip Chuanchuen

**Affiliations:** 1 Research Unit in Microbial Food Safety and Antimicrobial Resistance, Department of Veterinary Public Health, Faculty of Veterinary Science, Chulalongkorn University, Bangkok, Thailand; 2 Department of Veterinary Medicine, Faculty of Veterinary Science, Chulalongkorn University, Bangkok, 10330 Thailand; 3 Ministry of Education and Training, Faculty of Animal Science and Veterinary Medicine, Nong Lam University, Ho Chi Minh City, Vietnam; 4 Global Regulatory & Technical Advisor, Microbiology & Antimicrobials, Elanco Animal Health Inc, Basingstoke, England

**Keywords:** Antimicrobials, Bacteria, Microbial genetics

## Abstract

This study aimed to determine the percentage of colistin resistant and ESBL-producing *Escherichia coli* from clinically sick and healthy pigs and understand the molecular mechanisms underlying colistin resistance and ESBL production. A total of 454 *E. coli* isolates from healthy pigs (n = 354; piglets, n = 83; fattening pigs, n = 142 and sows, n = 100) and sick pigs (n = 100) were examined for antimicrobial susceptibility, chromosomal and plasmid-mediated colistin resistance mechanisms and ESBL genes. The healthy (41%) and sick pig (73%) isolates were commonly resistant to colistin. Three *mcr* genes including *mcr-1* (10.4%), *mcr-2* (1.1%) and *mcr-3* (45%) were detected, of which *mcr-3* was most frequently detected in the healthy (33%) and sick pig (57%) isolates. Coexistence of *mcr-1/mcr-3* and *mcr-2/mcr-3* was observed in piglets (23%), fattening pig (3.5%) and sick pig (13%) isolates. Three amino acid substitutions including E106A and G144S in PmrA and V161G in PmrB were observed only in colistin-resistant isolates carrying *mcr-3*. The percentage of ESBL-producing *E. coli* was significantly higher in the sick pigs (44%) than the healthy pigs (19.2%) (*P* = 0.00). The *bla*_CTX-M_ group was most prevalent (98.5%), of which *bla*_CTX-M-14_ (54.5%) and *bla*_CTX-M-55_ (42.9%) were predominant. The *bla*_TEM-1_ (68.8%) and *bla*_CMY-2_ (6.3%) genes were identified in ESBL-producers. All ESBL producers were multidrug resistant and the majority from piglets (97%), fattening pigs (77.3%) and sick pigs (82%) carried *mcr* gene (s). ESBL producers from piglets (n = 5) and sick pig (n = 1) simultaneously transferred *bla*_TEM-1_ (or *bla*_CTX-M-55_) and *mcr-3* to *Salmonella*. In conclusion, pigs are important reservoirs of colistin-resistant *E. coli* that also produced ESBLs, highlighting the need for prudent and effective use of antimicrobials in pigs and other food-producing animals.

## Introduction

In recent times antimicrobial resistance (AMR) has rapidly increased and become one of the greatest threats to public health globally. The highest-increasing rates of AMR have been reported in low and middle-income countries, especially those in Southeast Asia^1^. Extensive use of antimicrobials in either human medicine or animal farming is considered a major contributor to emergence and spread of AMR^2^. In livestock production, the purposes of antimicrobials are either to treat infections, control or promote growth^3^. Different countries have different policies and regulations with respect to antibiotic growth promoter (AGP). For example, Thailand phased in AGP ban in 2011 and implemented total ban in 2015^4^. The US FDA prohibited the use of medically important antibiotics for AGP in 2017 but not for non-medically important ones^5^. Consumer’s demand for livestock products has risen globally and is effectively driving antimicrobial consumption in food animals to maintain animal health and increase productivity. Some of these actions are consequently resulting in increasing levels of AMR^1^. The emergence of multi-drug resistant *E. coli* has been frequently reported not only in clinical medicine but also in livestock production. Particular concern has been raised to the dissemination of *E. coli* resistant to clinically important antibiotics (i.e. colistin, new generation cephalosporins and carbapenems) that may diminish antibiotics of choice for infection treatment in the near future.

Colistin is a cationic polypeptide antibiotic belonging to the class of polymyxins with a narrow antibacterial spectrum activity against certain Gram-negative bacteria. Although colistin is considered as a last resort antibiotic for treatment of serious infections caused by carbapenemase-producing Enterobacterales in human, its usage continues to be restricted due to its side effects (e.g. neurotoxicity and nephrotoxicity)^6^ and replaced by less toxic antibiotics, (e.g. aminoglycosides, quinolones, and β-lactams). In veterinary medicine, colistin has been commonly used in pig production for preventing and controlling the clinical outcomes of *E. coli* infection e.g. neonatal diarrhea, post-weaning diarrhea and edema disease by giving either in feed or in water^7^. However, its use in animals has been limited since 2016 as a consequence of the rising report of colistin resistance among the bacterial isolates from livestock, especially pig production^8^. Colistin resistance in *E. coli* can be associated with mutations in chromosomal genes i.e. *pmrA* and *pmrB*^9^. In 2016, the presence of transferable plasmid-mediated colistin resistance, *mcr-1,* was detected in Enterobacterales isolated from food animals, foodstuffs and humans in China and has posed a worrying threat to public health worldwide^8^. To date, several variants of plasmid-mediated colistin resistance genes (e.g. *mcr-2*, *mcr-3*, *mcr-4* and *mcr-5*) have been identified^10–12^. The *mcr-1* gene is globally distributed in many bacterial species isolated from various sources^6^. While *mcr-2* and *mcr-4* have been mainly identified in European countries^10,11^, *mcr-3* has been reported in *E. coli* from a variety of sources across Europe and Asia^12–15^. It is well noted that the *mcr-1* prevalence in bacteria isolated from food animals, especially swine, was higher than that from humans^8^.

ESBLs are enzymes conferring resistance to oxyimino cephalosporins (e.g. cefotaxime, ceftazidime and ceftriaxone) and oxyimino-monobactam aztreonam. Most ESBL encoding genes are located on conjugative plasmids^16^. ESBLs have been increasingly reported among Enterobacterales, particularly *E. coli* from food animals. The latter are considered an important reservoir of *E. coli* resistant to last-line antibiotics that can spread to humans via food chain.

ESBL-producing *E. coli* carrying *mcr-1* have been isolated from food animals and humans^17^. Co-existence of *mcr-1* with an ESBL gene on plasmids e.g. *bla*_VIM-1_^18^ and *bla*_CTX-M1_^19^) was previously demonstrated in clinical *E. coli* isolates. A former study in China reported the increasing prevalence of ESBL- producing *E. coli* in chicken origin from 2008 to 2014, of which the *mcr-1* gene was more prevalent in ESBL producers than non-ESBL-producers^17^. To date, many studies have investigated the emergence and dissemination of plasmids involved in colistin and cephalosporin resistance in livestock production and role of food animals as potential reservoirs of resistant bacteria and resistance genes was highlighted^17^. However, the knowledge of colistin and cephalosporin resistance associated with livestock in Asia, including Thailand, is still limited. Sick pigs are usually given antibiotic treatment and potentially contribute to emergence and spread of AMR. Only healthy animals are expected to be slaughtered for human consumption. They should not receive antibiotic therapy but may previously receive antibiotics for either growth promotion or disease prevention before slaughtering. Therefore, their role in AMR spreading cannot be overlooked. Only healthy pigs are expected to be slaughtered for human consumption and their commensal *E. coli* serve as reservoirs of AMR genes that may contaminate pork and pork products. Different AMR level is expected to be observed between the two groups of animals. Therefore, AMR monitor in both sick and healthy pigs is suggested. The aims of this study were to determine the percentage of ESBL production and colistin resistance and the distribution of ESBL and plasmid-mediated colistin resistance genes in *E. coli* isolated from clinically healthy as well as sick pigs.

## Materials and methods

### Bacterial isolates

A total of 454 *E. coli* isolates were obtained from two bacterial culture stocks isolated between 2007 and 2018 as described below. All *E. coli* strains were isolated by using standard method as previously described^20^. One *E. coli* colony from each positive sample was collected and stored in 20% glycerol at – 80 °C.

#### Isolates from healthy pigs

**Figure 1:**
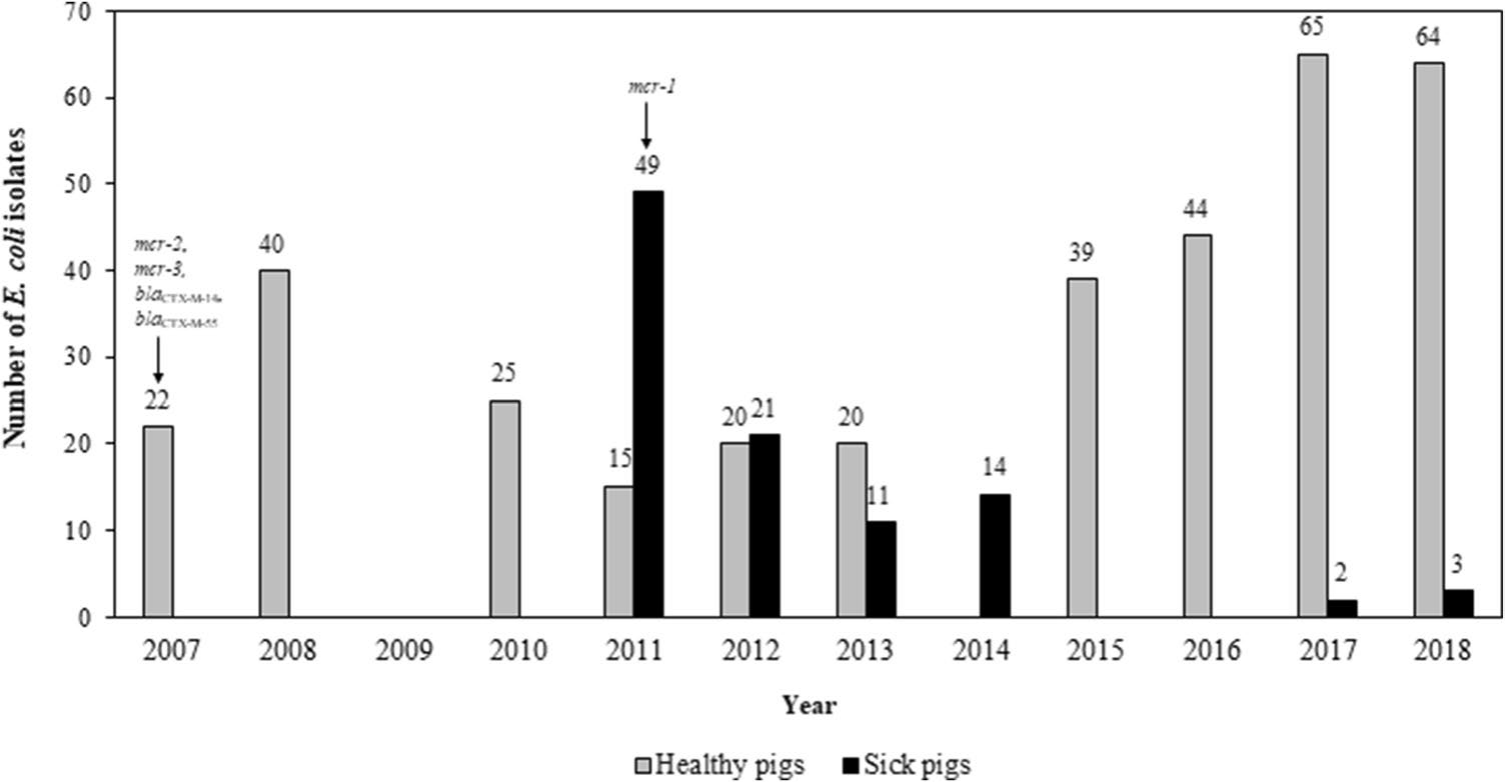
Yearly distribution of *Escherichia coli* from healthy pigs (n = 354) and sick pigs (n = 100) in Thailand between 2007 and 2018. The arrows indicates the first detection year of corresponded resistance genes.

Isolates were obtained from the bacterial stock of Department of Veterinary Public Health, Faculty of Veterinary Science, Chulalongkorn University (n = 354). These isolates originated from fecal samples collected from clinically healthy pigs, confirmed by farm veterinarians, piglets at 4–8 weeks of age (n = 83), fattening pigs at 9-18 weeks of age (n = 142) and sow at 37–45 weeks of age (n = 129) between 2007 and 2018 as part of our AMR studies. A yearly distribution of the isolates is shown in Fig. 1. The samples originated from farms located in Central and Northeast Thailand including Aungthong, Chachoengsao, Chonburi, Kanchanaburi, Ratchaburi, Suphanburi, Nakhonratchasima, Burirum, and Udonthani regions. These provinces have high densities of pig population, with farm sizes varying from small scale (51–500 pigs) to large scale (> 5000 pigs). Faecal samples were randomly collected from pigs of different age (one sample from one pig) by farm veterinarians. One isolate from each group of pigs at different age in each farm was used for antimicrobial susceptibility testing.

#### Isolates from sick pigs

The isolates were obtained from the strain collection of Department of Veterinary Medicine, Faculty of Veterinary Science, Chulalongkorn University (n = 100). All were isolated from fecal swab samples routinely collected from sick pigs at 2–21 weeks old displaying clinical signs of diarrhea during 2011–2018 (Fig. 1). Farm veterinarians collected and submitted samples for clinical diagnosis at Veterinary Diagnostic Laboratory (VDL), Livestock Animal Hospital, the Nakornpathom campus. The farms from which these samples were obtained were located in Central (i.e. Nakornpathom, Saraburi and Suphanburi), Eastern (i.e. Chachoengsao and Chonburi), Western (i.e. Kanchanaburi and Ratchaburi) and Southern (i.e. Trang) regions of Thailand. Antibiotic use history was not available.

### Test for antimicrobial susceptibility and extended spectrum β-lactamase (ESBL) production

Antimicrobial susceptibility was determined against 8 antimicrobial agents using the agar dilution method^21^. Antimicrobials tested, concentration ranges and clinical breakpoints, in parenthesis, are as follows: ampicillin (0.5–512 µg/mL, 32 µg/mL), chloramphenicol (0.5–512 µg/mL, 32 µg/mL), ciprofloxacin (0.125–256 µg/mL, 4 µg/mL), gentamicin (0.25–256 µg/mL, 8 µg/mL), streptomycin (0.5–1024 µg/mL, 32 µg/mL), sulfamethoxazole (0.5–1024 µg/mL, 512 µg/mL), tetracycline (0.5–512 µg/mL, 16 µg/mL) and trimethoprim (0.25–512 µg/mL, 16 µg/mL). Phenotypic resistance to colistin (0.25–128 µg/mL,) was tested by using two-fold agar dilution method^21^ and MIC results was interpreted according to European Committee on Antimicrobial Susceptibility Testing (EUCAST) breakpoints for Enterobacteriaceae (MIC > 2 µg/mL)^22^. *E. coli* ATCC® 25922, *Pseudomonas aeruginosa* ATCC® 27853, and *Staphylococcus aureus* ATCC® 29213 served as quality control strains.

Detection of ESBL production was performed by the disk diffusion method using antibiotics (quantity of antibiotic, zone diameter breakpoint) as follows: cefotaxime (30 µg,  ≤ 27 mm), cefpodoxime (10 µg, ≤ 17 mm) and ceftazidime (30 µg, ≤ 22 mm)^21^. The antibiotic disks were obtained from Oxoid (Oxoid™, Hamshire, England). *E. coli* ATCC® 25922 and *K. pneumoniae* ATCC® 700603 served as quality control strains.

The *E. coli* isolates exhibiting resistance to at least one cephalosporin tested were phenotypically confirmed for ESBL production using the combination disk method including cefotaxime and cefotaxime (30 mg)/clavulanic acid (10 mg), and ceftazidime and ceftazidime (30 mg)/clavulanic acid (10 mg) (Oxoid™, Hamshire, England). The inhibition zone difference of ≥ 5 mm in the combination with clavulanic acid versus the inhibition zone in the cephalosporin alone was interpreted as positive for ESBL production^21^.

### DNA isolation, PCR and DNA sequencing analysis

DNA template for PCR was prepared by whole cell boiled lysates as previously described^23^. All PCR amplifications were performed using TopTaq™ Master Mix Kit (QIAGEN, Germantown, MD, USA) according to the manufacturer’s instruction. Primers used in this study are listed in Supplementary Table 1. PCR products were separated on 1.5% agarose gel electrophoresis (Sigma-Aldrish®) in 1XTris-acetate/EDTA (TAE) buffer. The gels were stained in RedSafe™ Nucleic Acid Staining Solution (iNtRON Biotechnology, NJ, USA) and visualized using the Omega Fluor™ Gel Documentation System (APLEGEN™ Gel Company, CA, USA). The PCR products were purified using Nucleospin® Gel and PCR clean up (Macherey–Nagel, Düren, Germany) and submitted for DNA sequencing at First Base Laboratories (Selangor Darul Ehsan, Malaysia). The DNA sequences obtained were compared with the reference sequence available at GenBank Database using the Blast algorithm (http://www.ncbi.nlm.nih.gov).

#### Detection of mutations in the pmrAB system

Twenty colistin-resistant *E. coli* isolates from healthy pigs (n = 10) and sick pigs (n = 10) were arbitrarily selected (n = 20) for PCR amplification of *pmrA* and *pmrB* genes^9^. Five colistin-susceptible isolates from healthy pigs were included as control. The PCR amplicons were gel purified and submitted for DNA sequencing using PCR primers. DNA sequences were compared to those of *E. coli* K12 (U00096.2) available at GenBank database.

#### Detection of plasmid-mediated colistin resistance determinants and β-lactamase genes

All *E. coli* isolates (n = 454) were screened for the presence of *mcr* genes by PCR using specific primers, including *mcr-1* (MCR1-IF and MCR1-IR)^8^, *mcr-2* (MCR2-IF and MCR2-IR)^10^, *mcr-3* (MCR3-IF and MCR3-IR)^12^ *and mcr-4* (MCR4-IF and MCR4-IR)^11^. All the ESBL positive-isolates (n = 112) were examined for the presence of β-lactamase genes using specific primers, including *bla*_CTX-M_ (*bla*_CTX-M__FW and *bla*_CTX-M__RW), *bla*_PSE-M_ (*bla*_PSE-M__FW and *bla*_PSE-M__RW), *bla*_SHV_ (*bla*_SHV__FW and *bla*_SHV__RW), *bla*_TEM_ (*bla*_TEM__FW and *bla*_TEM__RW), *bla*_CMY-1_ (*bla*_CMY-1__FW and *bla*_CMY-1__RW) and *bla*_CMY-2_ (*bla*_CMY-2__FW and *bla*_CMY-2__RW)^16,24–26^.

The identification of *bla*_CTX-M_ groups was performed in all *bla*_CTX-M_ positive isolates by multiplex PCR using specific primers for CTX-M group1 (MultiCTXMGp1_FW and MultiCTXMGp1_RW), CTX-M group 2 (MultiCTXMGp2_FW and MultiCTXMGp2_RW), CTX-M group 8/25 (CTX-M group 8/25_FW and CTX-M group 8/25_RW) and CTX-M group 9 (CTX-M group 9_FW and CTX-M group 9_RW)^27,28^. All the isolates positive to CTX-M group 1 were further examined for *bla*_CTX-M15_ (CTX-M15_SFW and CTX-M15_SRW)^29^. The PCR amplicons of *bla*_TEM_ and *bla*_CTX-M_ were subjected to direct sequencing and their subtypes were analyzed by BLAST search.

### Conjugation experiments

Biparental filter mating method was performed to test transferability of *mcr* and ESBL genes. All the *E. coli* isolates carrying *mcr* and/or ESBL genes served as donors and the spontaneous rifampicin-resistant *Salmonella* Enteritidis (SE12Rif^R^, rifampicin MIC = 256 µg/ml), was used as recipient^30^. The *Salmonella* transconjugants were confirmed on Xylose Lysine Deoxycholate agar (Difco, MD, USA) containing 32 µg/mL rifampicin and an appropriate antibiotic (i.e. 100 μg/mL ampicillin, or 2 μg/mL colistin). Transfer of *mcr* and ESBL genes was confirmed by PCR as described above.

### Statistical analysis

Comparisons of the association between antimicrobial resistance phenotype and resistance encoding gene were performed by using Pearson's chi-squared test (*χ*^2^) (SPSS, version 22.0). A *p*-value of < 0.05 was considered statistically significant. Odds ratios with 95% confidence intervals (CIs) were calculated.

### Ethics statement

This study was conducted under the approval of the Institutional Animal Care and Use protocol of the Faculty of Veterinary Science, Chulalongkorn University, Bangkok, Thailand (IACUC # 1831065). I declare that all methods were carried out in accordance with relevant guidelines and regulations of the Institutional Biosafety Committee of the CU-VET, Chulalongkorn University (CU-VET-IBC # 1731038).

## Results

### Antimicrobial susceptibility

#### Healthy pigs

Overall, 78% of the *E. coli* isolates from healthy pigs (n = 384) were resistant to at least one antimicrobial agent tested (Table 1). Most isolates from fattening pigs (96.5%, 137/142) and all the isolates from piglets and sows were resistant to at least one antimicrobial agent tested. Concurrently, the majority of the isolates in this study including all piglet isolates, 99.2% of the sow isolates and 94.4% of the fattening pig isolates, were MDR (resistant to at least three antimicrobial agents in different classes). However, there was no significant difference of MDR proportion among the *E. coli* isolates from different groups of the healthy pigs. Overall, the percentage of colistin-resistant *E. coli* was 40.7%.

Colistin resistance was predominant among the piglet isolates (95.2%), followed by the isolates from fattening pigs (43.7%) and sows (2.3%). The colistin resistance rate in the piglet isolates was significantly higher than that in the sow and fattening pig isolates (*p* < 0.05).

**Figure 2:**
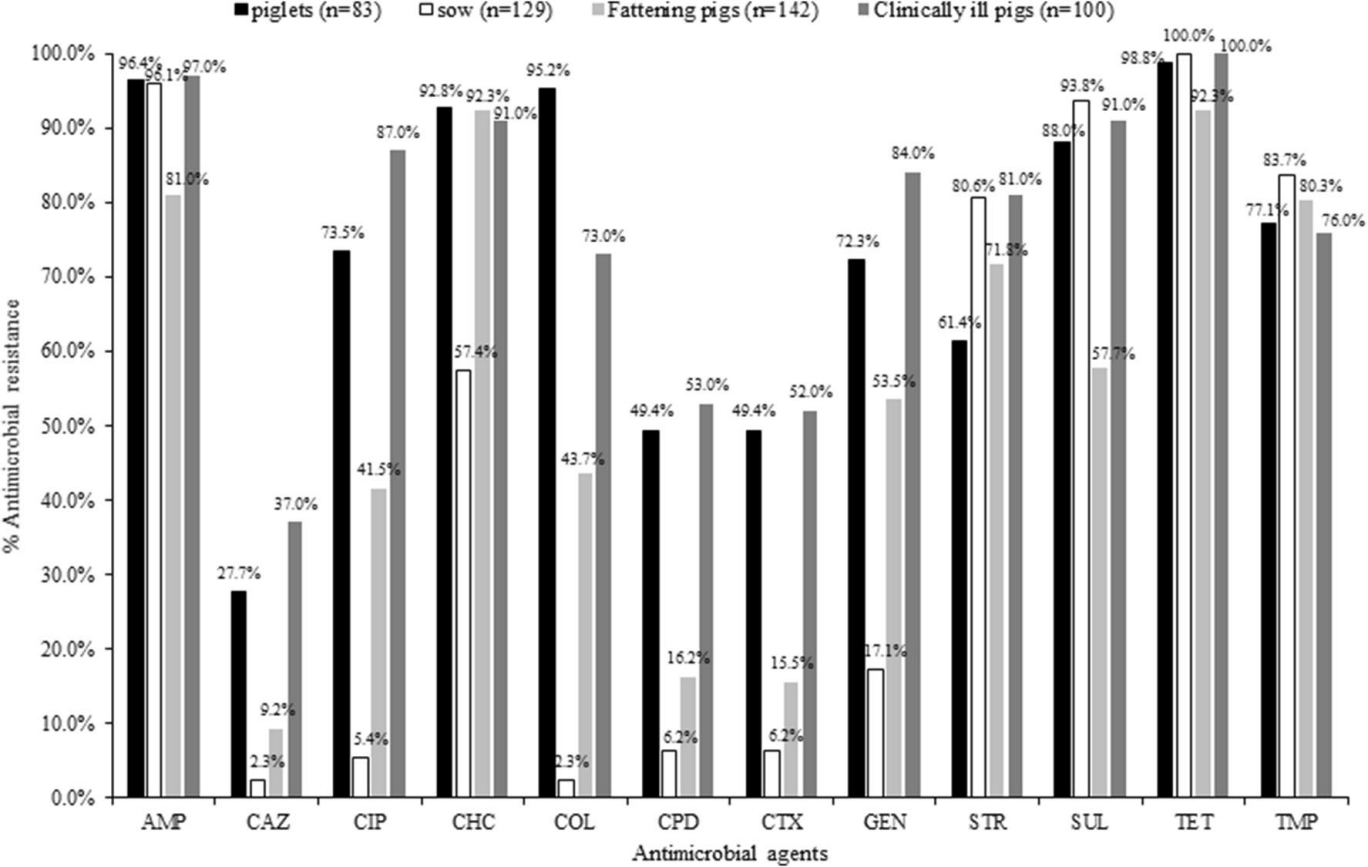
Distribution of antimicrobial susceptibility in *Escherichia coli* from clinically healthy pigs (n = 354) and sick pigs (n = 100) in Thailand between 2007 and 2018. *AMP* ampicillin, *CAZ* ceftazidime, *CIP* ciprofloxacin, *CHC* chloramphenicol, *COL* colistin, *CPD* cefpodoxime, *CTX* cefotaxime, *GEN* gentamicin, *STP* streptomycin, *SUL* sulfamethoxazole, *TET* tetracycline, *TMP* trimethoprim.

Sixty-eight (19.2%) *E. coli* isolates from the healthy pigs were confirmed to be ESBL-producers, including the isolates from piglets (45.8%, n = 38), fattening pigs (15.5%, n = 22) and sows (6.2%, n = 8). Resistance to ceftazidime, cefotaxime and cefpodoxime was highest in piglets (27.7%, 49.4%, 49.4%) followed by fattening pigs (9.2%, 15.5%, 16.2%) and sows (2.3%, 6.2%, 6.2%), respectively (Fig. 2). The percentage of ESBL-producing isolates was significantly higher in piglets than the others (*p* < 0.05). Similarly, the percentage of ESBL producers in fattening pigs was significantly higher when compared with that in sows (*p* < 0.05). The isolates from piglets, sows and fattening pigs were most frequently resistant to tetracycline (98.8%, 100%, 92.3%), ampicillin (96.4%, 96.1%, 81%) and chloramphenicol (92.8%, 57.4%, 92.3%), respectively (Fig. 2).

**Table 1:**
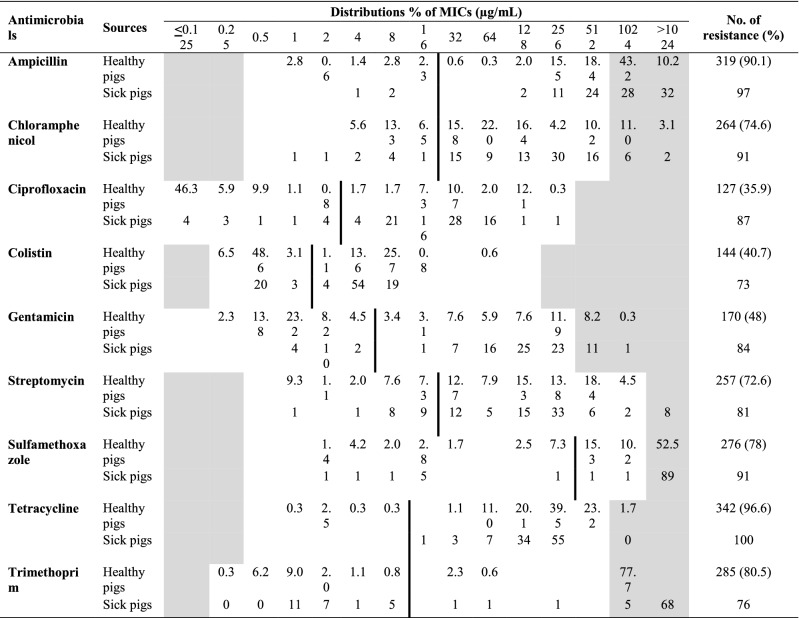
Distribution of Minimum Inhibitory Concentrations (MICs) and resistance percentages for the *E. coli* isolates from healthy pigs (n = 354) and sick pigs (n = 100).

White fields represent range of serial dilutions tested for each antimicrobial.

The MICs higher than the highest concentration tested are provided as the concentration closest above the range.

The clinical breakpoints for each antimicrobial are presented as a vertical line.

#### Sick pigs

All the *E. coli* isolates from sick pigs were resistant to at least one antimicrobial agent and up to 99% were MDR. Resistance to colistin was found in 73% of the isolates. Forty-four *E. coli* isolates (44%) in this group were ESBL-producers, that exhibited resistance to ceftazidime (53%), cefotaxime (53%) and cefpodoxime (37%). The percentage of ESBL-producing *E. coli* isolates was significantly higher in sick pigs than healthy pigs (*p* < 0.05). The majority of the sick pig isolates were resistant to tetracycline (100%) and ampicillin (97%) (Fig. 2).

### Presence and transfer of colistin resistance determinants

Of all the *E. coli* isolates tested (n = 454), the *mcr-1*(10.4%), *mcr-2* (1.1%) and *mcr-3* genes (45%) were identified. None of the isolates carried *mcr-4*.

Among the *E. coli* isolates from healthy pigs, the *mcr-1* (7.6%), *mcr-2* (1.4%) and *mcr-3* (37.9%) genes were detected. Four *mcr*-patterns were defined including, *mcr-1* (2.3%), *mcr-3* (31%), *mcr-1*/*mcr-3* (5.4%) and *mcr-2*/*mcr-3* (1.4%) (Table 2). Of all the isolates from healthy pigs in this collection, *mcr-1* was first detected in 2011, while *mcr-2* and *mcr-3* were found in 2007 at earliest (Fig. 1). Co-existence of *mcr-1/mcr-3* and *mcr-2*/*mcr-3* was detected in the isolates from piglet (23%) and fattening pig (3.5%), respectively. Colistin-MIC range was 8–64 µg/mL and 0.5–16 µg/mL for the isolates carrying only *mcr-1* and only *mcr-3*, respectively. The isolates harboring *mcr-3* in combination with *mcr-1* or *mcr-2* had colistin MIC of 4 or 8 µg/mL.

**Table 2:** Colistin resistance phenotype and genotype in *E. coli* isolates (n = 454) in Thailand between 2007 and 2018.

Source (n = 454)	No. colistin-resistant isolates (%)	Colistin resistance genotype		MIC (µg/mL)
		Genes	No. (% positive)	
**Healthy pigs**				
Piglets (n = 83)	79 (95.2)	*mcr-1*	7 (8.4)	8
		*mcr-3*	55 (66.3)	0.5–64
		*mcr-1/mcr-3*	19 (23)	4–8
Lactating sows (n = 129)	3 (2.3)	*mcr-1*	1 (0.8)	64
Fattening pigs (n = 142)	62 (43.7)	*mcr-2/mcr-3*	5 (3.5)	4–8
		*mcr-3*	55 (38.7)	0.5–16
**Sick pigs (n = 100)**	73 (73)	*mcr-1*	7 (7)	4–8
		*mcr-3*	57 (57)	1–8
		*mcr-1/mcr-3*	13 (13)	4–8
**Total**	217 (47.8)		219 (48.2)	

Among the *E. coli* isolates from sick pigs (n = 100), the *mcr-1* (20%) and *mcr-3* (70%) genes were found. Three *mcr* patterns including *mcr-1* (7%), *mcr-3* (57%) and *mcr-1/mcr-3* (13%) were observed. The colistin MIC of the isolates carrying *mcr-1* only was 4–8 µg/mL, while that of the isolates with *mcr-3* only was 1–8 µg/mL. The isolates with *mcr-1/mcr-3* had a colistin MIC of 4 or 8 µg/mL.

Based on the conjugation experiment in all *mcr*-positive *E. coli* (n = 219), *mcr-3* in two isolates (one from a piglet and one from a sick pig) was horizontally transferred to *Salmonella*. All the *Salmonella* transconjugants were confirmed to carry *mcr-3* and their colistin MIC was 4 µg/mL.

### Amino acid alterations in pmrAB

In comparison to *E. coli* K12, sequence variations in *pmrAB* were found in all *E. coli* tested (n = 25) (Table 3). Four amino acid substitutions including S29G, E106A, G144S and E184D were identified in PmrA and five amino acid substitutions including H2R, V161G, D283G, Y358N, A360V were detected in PmrB. The amino acid changes S29G in PmrA and H2R, D283G and Y358N in PmrB were found in both colistin-resistant and susceptible *E. coli* isolates. Among the healthy pig isolates, two *mcr-3* carrying isolates (i.e. E.453 and E.454) carried G144S amino acid substitution in PmrA and additionally harbored V161G in PmrB. The colistin MIC of both isolates was 16 µg/mL. One sick pig isolate (i.e. EC.P.45, colistin MIC = 8 µg/mL) carried both *mcr-3* and E06A amino acid substitution in PmrA. A colistin-susceptible isolate (i.e. GCa13, colistin MIC = 0.25 µg/mL) harbored E184D amino acid substitution in PmrA that was not observed in any colistin-resistant isolates tested.

**Table 3:** The presence of *mcr* genes and amino acid substitution in PmrAB among colistin-resistant isolates from pigs (n = 25) in Thailand between 2007 and 2018.

Sources	Isolates	COL MIC (µg/mL)	*mcr* gene	PmrA	PmrB
**Colistin resistant isolates**					
Healthy pigs	E.400^a^	8	*mcr-3*	AGC → GGC (S29G)	
	E.453^a^	16	*mcr-3*	AGC → GGC (S29G), GGC → AGC (G144S)	GTG → GGG (V161G)*,* GAC → GGC (D283G), TAC → AAC (Y358N)
	E.454^a^	16	*mcr-3*	AGC → GGC (S29G), GGC → AGC (G144S)	GTG → GGG (V161G)*,* GAC → GGC (D283G)*,* TAC → AAC (Y358N)
	E.458^a^	8	*mcr-3*	AGC → GGC (S29G)	–
	E.459^a^	8	*mcr-3*	AGC → GGC (S29G)	–
	PLEa 3^b^	64	*mcr-3*	AGC → GGC (S29G)	–
	LCa 7^c^	64	*mcr-1*	–	GAC → GGC (D283G), TAC → AAC (Y358N)
	PLEa 26^b^	8	*mcr-3*	AGC → GGC (S29G)	–
	FPEa 13^b^	8	*mcr-1*	AGC → GGC (S29G)	–
	FPEa 19^b^	8	*mcr-1*	AGC → GGC (S29G)	–
Sick pigs	EC.P. 5	4	*mcr-1, mcr-3*	AGC → GGC (S29G)	GAC → GGC (D283G)*,* TAC → AAC (Y358N)
	EC.P. 9	4	*mcr-1*	AGC → GGC (S29G)	CAT → CGT (H2R)*,* GAC → GGC (D283G)
	EC.P. 10	4	*mcr-1*	AGC → GGC (S29G)	GAC → GGC (D283G), TAC → AAC (Y358N)
	EC.P. 16	4	*mcr-1*	AGC → GGC (S29G)	GAC → GGC (D283G), TAC → AAC (Y358N)
	EC.P. 40	4	*mcr-1, mcr-3*	AGC → GGC (S29G)	–
	EC.P. 45	8	*mcr-3*	AGC → GGC (S29G), GAA → GCA (E106A)	–
	EC.P. 46	4	*mcr-3*	AGC → GGC (S29G)	–
	EC.P. 47	4	*mcr-3*	AGC → GGC (S29G)	–
	EC.P. 48	4	*mcr-3*	AGC → GGC (S29G)	–
	EC.P. 49	8	*mcr-3*	AGC → GGC (S29G)	–
**Colistin susceptible isolates**	LCa 6^d^	0.25	–	AGC → GGC (S29G)	–
	LCa 9^d^	0.25	–	AGC → GGC (S29G)	GAC → GGC (D283G), TAC → AAC (Y358N)
	LCa 10^d^	0.25	–	AGC → GGC (S29G)	CAT → CGT (H2R)*,* GAC → GGC (D283G)*,* GCA → GTA (A360V)
	GCa 12^d^	0.25	–	AGC → GGC (S29G)	GAC → GGC (D283G)*,* TAC → AAC (Y358N)
	GCa 13^d^	0.25	–	AGC → GGC (S29G), GAA → GAC (E184D)	*-*

^a^Fattening pigs, ^b^Piglets, ^c^Lactating and ^d^Gestating sows.

### Presence and transfer of β-lactamase genes

One-hundred twelve ESBL-producing *E. coli* were screened for β-lactamase genes. The majority of ESBL-producing isolates were positive to *bla*_CTX-M_ (98.5%), of which the majority were CTX-M group 9 (54.5%), followed by CTX-M group 1 (42.9%) (Table 4). The CTX-M group 2 and CTX-M group 8/25 were not detected. DNA sequencing analysis confirmed that all CTX-M group 9 (n = 61) were *bla*_CTX-M-14_ and all CTX-M group 1(n = 48) were *bla*_CTX-M-55_. All *bla*_TEM_ were confirmed to be *bla*_TEM-1_ and were observed in 53 (78%) isolates from piglets (n = 32), sows (n = 7) and fattening pigs (n = 14). Twenty-seven of *bla*_CTX-M-55_ (90%) and 28 of *bla*_CTX-M-14_ (75.7%) positive isolates simultaneously carried *bla*_TEM-1_. The *bla*_CMY-2_ gene was found in two isolates from sows. One of the *bla*_CMY-2_ -positive isolate harbored *bla*_TEM-1_ and *bla*_CTX-M-14_ (CMY2/TEM-1/CTX-M14/), while the others additionally carried *bla*_TEM-1_ and *bla*_CTX-M-55_ (CMY-2/TEM-1/CTX-M55). The first detection of *bla*_CTX-M-14_ and *bla*_CTX-M-55_ was in the *E. coli* isolates from fattening pigs in 2007. These isolates additionally carried *bla*_TEM-1_.

**Table 4:** Distribution of β-lactamase genes among *Escherichia coli* isolates from healthy and sick pigs (n = 454) in Thailand between 2007 and 2018.

β-lactamase genotype pattern	Healthy pigs [no. of isolates (%)]			Sick pigs (n = 44)	Total (n = 112)
	Piglets (n = 38)	Sows (n = 8)	Fattening pigs (n = 22)		
TEM-1	1 (1.2)	–	1 (0.7)	2 (2)	4 (3.6)
CTX-M-55	1 (1.2)	1 (0.8)	–	9 (9)	11 (9.8)
CTX-M-14	3 (3.6)	–	5 (3.5)	10 (10)	18 (16)
CTX-M-55, CTX-M-14	1 (1.2)	–	–	1 (1)	2 (1.8)
TEM-1, CTX-M-55	17 (20.5)	2 (1.6)	7 (4.9)	7 (7)	33 (29.5)
TEM-1, CTX-M-14	15 (18)	3 (2.3)	9 (6.3)	10 (10)	37 (33)
TEM-1, CMY-2	–	–	–	1 (1)	1 (0.9)
TEM-1, CTX-M-55, CMY-2	–	1 (0.8)	–	1 (1)	2(1.8)
TEM-1, CTX-M-14, CMY-2	–	1 (0.8)	–	3 (3)	4 (3.6)
Total	38 (45.8)	8 (6.2)	22 (15.5)	44 (44)	112 (24.7)

Among the ESBL-producing *E. coli* isolates from sick pigs (n = 44), only *bla*_CTX-M55_ (41%) and *bla*_CTX-M14_ (54.5%) were identified. The *bla*_TEM-1_ gene was found in 54.5%, of which 10 isolates co-existed with *bla*_CTX-M-14_ and 7 isolates co-existed with *bla*_CTX-M-55_. The *bla*_CMY-2_ gene was identified in 4 isolates, of which 3 isolates simultaneously carried *bla*_CTX-M-14_ and *bla*_TEM-1_ and one isolate carried *bla*_CTX-M-55_ and *bla*_TEM-1_.

### Co-existence of ESBL and mcr genes

Up to 90 isolates (80.4%) of ESBL producers in this study (n = 112) additionally harbored *mcr* genes, of which nearly 50% (n = 54) originated from healthy pigs (piglets, n = 37; fattening pigs, n = 17) and 32% (n = 36) were from sick pigs. Four *mcr* patterns of these ESBL producers were identified including *mcr-1* (3.6%)*, mcr-3* (62.5%), *mcr-1*/*mcr-3* (9.8%) and *mcr-2*/*mcr-3* (4.5%). The *mcr-3* gene was most commonly observed in ESBL producers (76.8%) (Table 5).

**Table 5:** The presence of *mcr* and β-lactamase genes in *E. coli* from healthy and sick pigs (n = 454) in Thailand between 2007 and 2018.

Origins	Sources^a^	Colistin resistance gene^a^	Β-lactamase gene^a^
Healthy pigs	Piglets (37)	*mcr-1* (2)	*bla*_TEM-1_ and *bla*_CTX-M-55_ (2)
		*mcr-1/mcr-3* (5)	*bla*_TEM-1_ and *bla*_CTX-M-55_ (5)
		*mcr-3* (30)	*bla*_CTX-M-14_ (3) *bla*_CTX-M-55_ (1) *bla*_CTX-M-55_ and *bla*_CTX-M-14_ (1) *bla*_TEM-1_ and *bla*_CTX-M-55_ (10) *bla*_TEM_-_1_ and *bla*_CTX-M-14_ (15)
	Fattening pigs (17)	*mcr-2/mcr-3* (5)	*bla*_CTX-M-14_ (5)
		*mcr-3* (12)	*bla*_TEM-1_ and *bla*_CTX-M-55_ (9) *bla*_CTX-M-14_ (3)
Sick pigs	Pig age 2–21 weeks (36)	*mcr-1* (2)	*bla*_TEM-1_, *bla*_CTX-M-55_ and *bla*_CMY-2_ (1) *bla*_CTX-M-14_ (1)
		*mcr-1/mcr-3* (6)	*bla*_TEM-1_ and *bla*_CTX-M-55_ (1) *bla*_TEM_-_1_ and *bla*_CTX-M-14_ (2) *bla*_CTX-M-55_ and *bla*_CTX-M-14_ (1) *bla*_TEM-1_, *bla*_CTX-M-55_ and *bla*_CMY-2_ (1) *bla*_TEM-1_, *bla*_CTX-M-14_ and *bla*_CMY-2_ (1)
		*mcr-3* (28)	*bla*_CTX-M-55_ (9) *bla*_CTX-M-14_ (7) *bla*_TEM-1_ and *bla*_CTX-M-55_ (6) *bla*_TEM-1_ and *bla*_CTX-M-14_ (5) *bla*_TEM-1_, *bla*_CTX-M-14_ and *bla*_CMY-2_ (1)

^a^Numbers in parenthesis indicate the number of positive *E. coli* isolate(s).

Horizontal transfer of β-lactamase genes was observed in 14 *E. coli* isolates. Five piglet isolates transferred *bla*_TEM-1_ and co-transferred *mcr-3*. One sick pig isolate was capable of transferring *bla*_CTX-M55_ and *mcr-3* simultaneously. Seven *E. coli* isolates including 2 isolates from piglets (one isolates with *bla*_CTX-M-14_ and the others with *bla*_CTX-M-55_) and 4 isolates from sick pigs (2 isolates with *bla*_CTX-M-14_ and 2 isolates with *bla*_CTX-M-55_) were able to transfer *bla*_CTX-M_. One isolate from sick pig could transfer both *bla*_CTX-M-55_ and *bla*_TEM-1_ gene at the same time.

### Association between AMR phenotype and genotype

The associations between resistance phenotype and genotype varied (Table 6). The positive associations between resistance phenotype and the presence of *mcr* or β-lactamase genes were as follows: CIP resistance/CTX-M-14; STR resistance/*mcr-1*, CTX-M-55; SUL resistance/*mcr-2*, TEM-1, CTX-M-55; TET resistance/*mcr-2*, TEM-1, CTX-M-14, CTX-M-55 and TMP resistance/*mcr-1*, *mcr-2* and CTX-M-14. The strongest positive association was observed between TET and CTX-M-55 (OR = 31, 8.05–119.3) and TET and *mcr-2* (OR = 9.95, 1.02–96.5).

**Table 6:** Associations between resistance phenotype and genotype in *Escherichia coli* from healthy pigs and sick pigs (n = 454) in Thailand between 2007 and 2018.

ABO resistance gene (n)	AMP		CAZ		CIP		CHC		COL		CPD		CTX		GEN		STR		SUL		TET		TMP	
	No^a^	*Assoc*^*b*^	No	*Assoc*	No	*Assoc*	No	*Assoc*	No	*Assoc*	No	*Assoc*	No	*Assoc*	No	*Assoc*	No	*Assoc*	No	*Assoc*	No	*Assoc*	No	*Assoc*
*mcr-1* (46)	45	0.2 (0.03–1.6)	15	0.4 (0.2–0.7)	36	0.22 (0.1–0.45)	41	0.4 (0.16–1.06)	46	–	16	0.68 (0.36–1.3)	16	0.67 (0.35–1.3)	34	0.37 (0.19–0.7)	30	1.64 (0.86–3.14)	41	0.49 (0.19–1.27)	46	–	23	4.91 (2.6–9.3)
*mcr-2* (5)	5	–^c^	1	0.8 (0.1–7.3)	5	–	5	–	5	–	5	–	5	–	5	–	5	–	4	1.05 (0.12–9.6)	4	9.95 (1.02–96.5)	3	2.65 (0.44–16.2)
*mcr-3* (204)	197	0.3 (0.1–0.6)	65	0.09 (0.1–0.2)	159	0.08 (0.05–0.12)	185	0.2 (0.13–0.38)	193	0.006 (0.003–0.013)	100	0.12 (0.07–0.19)	104	0.08 (0.05–0.14)	154	0.18 (0.12–0.27)	156	0.8 (0.54–1.26)	173	0.62 (0.38–1.0)	201	0.4 (0.11–1.5)	166	0.83 (0.52–1.3)
TEM-1 (81)	77	0.5 (0.2–1.5)	46	0.7 (0.04–0.12	59	0.27 (0.16–0.45)	72	0.39 (0.19–0.8)	62	0.2 (0.13–0.38)	76	0.01 (0.00–0.03)	76	0.009 (0.00–0.03)	73	0.09 (0.04–0.19)	64	0.74 (0.4–1.3)	62	1.38 (0.77–2.45)	75	4.9 (1.5–15.6)	68	0.7 (0.4–1.4)
CTX-M-14 (61)	59	–	12	0.04 (0.02–0.08)	47	2.6 (1.4–4.7)	45	1.3 (0.7–2.5)	44	0.03 (0.17–0.5)	58	0.01 (0.00–0.04)	58	0.01 (0.00–0.03)	55	0.09 (0.04–0.24)	51	0.53 (0.26–1.08)	53	0.6 (0.27–1.3)	57	3.37 (0.98–11.6)	43	1.8 (0.98–3.3)
CTX-M-55 (48)	48	–	48	0.18 0.08–0.4)	39	0.18 (0.08–0.4)	48	-	46	0.03 (0.01–0.13)	48	–	48	–	48	–	35	1.09 (0.56–2.15)	38	1.12 (0.54–2.36)	49	31 (8.05–119.3)	46	0.15 (0.04–0.6)
CMY-2 (7)	7	–	6	0.35 (0.07–1.8)	5	0.35 (0.07–1.8)	6	0.59 (0.07–4.9)	5	0.36 (0.07–1.9)	7	–	7	–	6	0.19 (0.02–1.56)	7	–	7	–	7	–	6	0.65 (0.07–5.5)

^a^No., number of isolates resistant to corresponding antimicrobial agents and carrying the relevance resistance genes.

^b^Odds ratio (OR) for significant associations between antimicrobial resistance gene and antimicrobial resistance phenotype (95% confidence interval in parenthesis). OR > 1 represents positive associations, and OR < 1 represents negative associations.

^c^No significant associations (*P* ≥ 0.05).

*AMP* ampicillin, *CAZ* ceftazidime, *CIP* ciprofloxacin, *CHC* chloramphenicol, *COL* colistin, *CPD* cefpodoxime, *CTX* cefotaxime, *GEN* gentamicin, *STP* streptomycin, *SUL* sulfamethoxazole, *TET* tetracycline, *TMP* trimethoprim.

## Discussion

The present study was conducted in *E. coli* isolates from clinically healthy and clinically sick pigs collected during the time period 2007–2018. One significant finding was high MDR rates in the isolates from healthy (97.5%) and sick pigs (99%). It is expected that only healthy pigs are slaughtered for human consumption, but their health status does not guarantee the absence of resistant bacteria. This is because antibiotics may be previously administered to the pigs that were the source of the isolates for disease prevention and growth promotion, which may have resulted in commensal bacteria developing antibiotic resistance. Similarly, carrying resistant bacteria does not infer having a disease. As the complete ban of AGP in all animal feed was implemented in 2015, use of AGP could influence the high AMR rates observed in earlier years in this study. In consideration of the dynamics of AMR, antibiotic administration and AMR development may not simultaneously occur. At the same time, it is still unclear to what extent antibiotic use must be reduced, and how long the interventions must be made to effectively to reverse the spread of AMR.

The highest frequency of resistance among the isolates from healthy and sick pigs was to tetracycline and ampicillin, in agreement with a previous study in *E. coli* isolated from pig farms in Thailand^31^. However, it was not possible to obtain the antibiotic use history in each farm. The antibiotics are usually administered to piglets by oral route either in feed or in water for controlling gastrointestinal tract infection in piglets including polypeptides (e.g. colistin) and aminoglycosides (e.g. apramycin). Tylosin, tilmicosin and chlortetracycline were used in fattening pigs. Cephalosporins (e.g. ceftiofur and ceftriaxone) are occasionally used for treatment of respiratory diseases, lameness, and reproductive infections. It was estimated that approximately 39.7% of medicated feed was used in suckling and nursery pigs followed by fattening pigs (37.3%) and breeding pig (23%) in Thailand^7^. Some antibiotics mixed in medicated feed used in pig production in the country are included in WHO list of Critically Important Antimicrobials for Human Medicine e.g. amoxicillin, colistin and lincomycin^7,32^. Up to date, Thailand has launched law and regulations to contain AMR associated with food animals, for example, Notification of the Ministry of Agriculture and Cooperatives that specifically prohibits the use of all antibiotics in animal feed as growth promoters was released in 2015^4^. Law on “Characteristics and conditions of animal feed containing drugs prohibited from producing, importing, selling and using” was issued in 2018, of which medicated feed containing polymyxin B, cephalosporins, fluoroquinolones and others are covered by this law^33^. A year later, regulation of antimicrobial drugs that must not be mixed in animal feed for prophylactic purposes was announced^34^. The latter included polymyxins B, colistin and other drugs in penicillin and fluroquinolone groups. Effective enforcement of these regulations is expected and the outcomes of implementation may be seen through national AMR surveillance data in coming years.

A note could be made for ciprofloxacin resistance (35.9% for the healthy pig isolates and 87% for the sick pig isolates) that was defined by clinical breakpoints used. Being ciprofloxacin susceptible does not always warrant being wildtype lacking alterations in target fluoroquinolone genes. The different contribution of a certain amino acid substitution to fluroquinolone resistance was previously suggested^35^. However, interactions with target site mutations and ciprofloxacin resistance level were not pursued in this study.

Isolates from healthy (40.7%) and sick pigs (73%) exhibited a high colistin resistance rate that was higher than in a previous study conducted in *E. coli* from healthy and diseased pigs in Japan between 2012–2013^14^. The highest colistin resistance rate was found in the isolates from piglets (95.2%), followed by sick pigs (77%). This is likely because colistin has been used for treatment of gastrointestinal tract infections caused by *E. coli*, especially in post-weaning diarrhea in piglets^7^. Colistin was commonly formulated into medicated feed for suckling and nursery pigs for the prevention of gastrointestinal tract infection in Thailand^7^. Approximately 40 tons of colistin were mixed in medicated feed and about 87.2% were intended for piglets in Thai pig production^7^. Such extensive use of colistin may contribute to high colistin resistance rate observed in the present study. Implementation to minimize use and encourage prudent use of colistin and other antimicrobials is mandatory. In addition, the colistin resistance rates in the piglets (95.2%) and sow (2.3%) isolates were quite different. Piglets usually acquire intestinal flora including *E. coli* from the mother at birth and therefore, the similar resistance rates are expected in the piglets and sow isolates. The discrepancy observed in this study could be attributed to different pattern of colistin administration to pigs at the different stages. Colistin is most often administered orally in medicated feed or individually by a feeding bottle to suckling piglets and nursery pigs to treat post-waning diarrhea (PWD) and colibacillosis^7^. However, this is not the case for sows. Another explanation could be involved in the sources of the isolates, of which the piglet and sow isolates were obtained from several studies in different years and from different pig farms with different pattern of antimicrobial usage.

Chromosomal mutations in the two-component regulatory system of PmrAB were previously shown to be significantly associated with colistin resistance in bacterial pathogens such as *Klebsiella pneumoniae* and *Salmonella enterica*, *Acinetobacter baumannii* and *Pseudomonas aeruginosa*^36^. However, mutations in PmrAB is rarely reported in *E. coli*. A previous study demonstrated amino acid substitutions S39I and R81S in PmrA and V161G in PmrB in colistin-resistant *E.coli* isolates from pigs in Spain^9^. However, the S39I and R81S amino acid substitutions in PmrA were not found in this study. In addition to mobile colistin resistance (*mcr*) genes, research studies focusing on the chromosomal-mediated colistin resistance and their regulatory mechanism have increased^36^. Some mutations (i.e. E106A and G144S in PmrA and V161G in PmrB) were observed only in colistin-resistant isolates carrying *mcr-3* in this study. However, individual contribution and cumulative effects of the genes to colistin resistance was not determined and needs further investigations. At the same time, some amino acid changes (e.g. S29G in PmrA and D283G, Y358N and H2R in PmrB) were identified in both colistin-resistant and colistin-susceptible isolates, suggesting the lack of impact on colistin resistance phenotype. Studies of other TCSs and their regulators such as PhoPQ, MgrB, and PmrD are suggested^36^.

In this study, *mcr-3* was most predominant among the *E. coli* isolates from both healthy pigs (32.5%) and sick pigs (57%), while the lower percentage of *mcr-1* was observed in healthy pigs (7.6%) and in sick pigs (20%). These results are inconsistent to a previous study reporting that *mcr-1* was commonly detected in *E. coli* from healthy and diseased pigs in Japan (45%) and *mcr-3* was found at lower rate (8.3%) in diseased pigs^14^. The discrepancies may be due to difference in antimicrobial usage patterns or in the prevalence of different clones and/or plasmids.

The *mcr-1* gene is globally distributed and has been found in many bacterial species (e.g. *E. coli*, *Salmonella* spp*., Klebsiella* spp*.* and *Pseudomonas* spp*.*) from food animals, food stuff and human^8^. To date, *mcr-1* is commonly screened in colistin-resistant isolates. Therefore, *mcr-1* in colistin-susceptible isolates and other *mcr* variants may be overlooked. Currently, there are still only limited report of *mcr-3*. Previous studies reported in the presence of *mcr-3* in cattle from France and Spain^15^, pigs and chicken from China^13^ and pigs from Japan^14^. However, *mcr-3* appear to be common among healthy and sick pigs in this study. Further studies in different animal sources and other countries should be conducted to determine the role of this gene in the dissemination of colistin resistance. Moreover, a previous study showed that *mcr-3* was commonly located on broad-host range plasmids (i.e. IncP) and several transposases and IS elements (i.e. IS*4321*, ΔTn*As2* and ISK*pn40*) were identified in the flanking regions of *mcr-3*. This might cause wider spread and stronger transmission capabilities of *mcr-3* than *mcr-1*^37^. Further genetic characterization of *mcr-3* carrying plasmid are needed to elucidate molecular mechanisms underlying dissemination of this gene*.*

The *mcr-2* positive isolates were detected (n = 5) in fattening pigs. The *mcr-2* gene was previously reported in colistin-resistant *E. coli* from pigs in Belgium (20.8%)^10^ and China (56.3%)^13^. None of the isolates in this study carried *mcr-4*. Up to date, the report of *mcr-4* has been limited to EU countries including *Salmonella* from pigs in Italy and *E. coli* from pigs in Spain and Belgium^11^. These variations suggest that spread and evolution of *mcr* genes should be monitored.

Coexistence of different *mcr* variants was observed, including *mcr-1*/*mcr-3* (23% of piglets and 13% of sick pigs) and *mcr-2/mcr-3* (3.5% of fattening pigs). The *E. coli* carrying *mcr-1/mcr-3* were previously isolated from cattle in Spain, pig and poultry in China and humans in New Zealand^13,15^. The isolates carrying both *mcr-1* and *mcr-2* were previously identified in pigs in Canada^38^. By considering the colistin MIC, all *mcr-1* harboring isolates exhibited resistance to colistin (colistin MIC 4–64 µg/mL). However, *mcr-3* can be found in colistin susceptible strains (colistin MIC 0.5–2 µg/mL), in agreement with a previous study^14^. In addition, all the *E. coli* isolates harboring more than one *mcr* genes had colistin MIC of 4 or 8 µg/mL. Taken together, the observations indicate that the number of *mcr* derivatives is not always related to colistin resistance level. As the contribution of individual *mcr* genes, especially *mcr-3*, to colistin resistance level remains to be elucidated, monitoring *mcr* variants should be conducted in colistin-susceptible and resistant strains.

The ESBL *E. coli* of healthy pig origin (19.2%) in this study was less common than that in a previous report in the isolates obtained during 2012–2013 in the same country^39^. The presence of ESBL producers in sick pigs (44%) was significantly higher than that in healthy pigs (*p* < 0.05). Among the healthy pigs, the highest percentage of ESBL producers was observed in piglets (45.8%) (*p* < 0.05). This is presumably associated with the common use of β-lactam antibiotics (e.g. amoxicillin and third-generation cephalosporins) in the suckling period for treatment of respiratory disease as suggested by a study of antimicrobial use in pigs in Germany^40^. The percentage of ESBL-producing *E. coli* in sick pigs (44%) was significantly higher than that in healthy pigs (19.2%) (*p* < 0.05). This may be a result of antibiotics previously administered to treat sick pigs. Cephalosporins are generally more expensive than other antimicrobial agents and may not be commonly used in pig production in Thailand and other countries in South East Asia. Currently, cephalosporins are increasingly used in pig production due to its long-lasting potency and lower doses. However, the presence of ESBLs may be also a result of other antimicrobial usage. This is because ESBL genes commonly colocalize on the same plasmid as other resistance genes.

The *bla*_CTX-M_ gene was the most prevalent ESBL gene in this study, in agreement with previous reports in Thailand^41^ and other countries in Asia e.g. China, Vietnam and India^29,42^. The majority of CTX-M subgroup was *bla*_CTX-M-14_ of CTX-M Gr.9 (54%), followed by *bla*_CTX-M-55_ of CTX-M Gr.1 (43%), in agreement with a previous study in livestock and environment in Thailand^41^.

Currently, the *bla*_CTX-M-55_ gene has been increasingly reported especially in China where *bla*_CTX-M-55_ is the second most frequent CTX-M variant in food-producing animals^17^. The *bla*_CTX-M-55_ gene was first identified in Thailand in 2005 among ESBL-producing *E. coli* obtained from human and then was identified in clinical isolates in other countries i.e. *K. pneumonia* in China and *Salmonella* spp. in the US^17^. Previous studies reported that *bla*_CTX-M-55_ was the major CTX-M subtype in ESBL-*E. coli* isolates from clinical isolates, food animals, farm waste and canals in Thailand^41^. The gene was predominant in *E. coli* from livestock and pets in other Asian countries e.g. China and Hong Kong^42^. The *bla*_CTX-M55_ gene was also detected in countries outside Asia but to less extent.

The β-lactamase gene, *bla*_TEM-1_ (72.3%) was commonly identified in this study. The gene has been frequently detected in the *E. coli* isolates from animals and is commonly co-harbored with ESBL genes^26^. This is in agreement with the current study where most ESBL producers (67.9%) carried TEM-1 and ESBL genes. The *bla*_CMY-2_ gene was detected at low frequency (5.4%). The gene was firstly identified in *K. pneumoniae* from human isolates and is increasingly reported in different bacteria from livestock e.g. *E. coli* from ground chicken and pig feces in Taiwan^43^, and *E. coli* from healthy chicken and sick animals in Spain^44^, in agreement with this study. In addition, the isolates carrying *bla*_CMY-2_ coharbored *bla*_CTX-M-55_ and *bla*_CTX-M-14_, in agreement with previous studies^45^.

Most ESBL producers from piglets (97%), fattening pigs (77.3%) and sick pigs (82%) additionally carried *mcr* genes, of which the most common *mcr* gene among ESBL producers was *mcr-3*. However, a previous study in China showed that *mcr-1* was more commonly found in ESBL *E. coli* than non ESBL producers^17^. β-lactams and colistin are bactericidal antibiotics that disrupt the outer membrane of bacterial cells. Recruiting *mcr* genes in the cell is a survival mechanism to maintain the cell wall integrity and may contribute to the increasing prevalence of ESBL producers coharboring *mcr* genes^17^. In addition, all ESBL-*mcr* carrying isolates were MDR, in agreement with a previous study^17^. These results highlight the continued need to encourage the prudent and effective use of antimicrobials in food animal production.

By using ampicillin as selectable marker, co-transfer of β-lactamase genes (*bla*_TEM-1_ and *bla*_CTX-M55_) and *mcr* gene (*mcr-3*) was detected, suggesting co-resistance of the gene on the same plasmid. This also suggest that distribution of *mcr* and ESBLs genes can be a result of co-selection by antibiotics in other classes.

In this study, the strength of the association between AMR phenotype and genotype was quantified. Strong positive correlation suggests possible genetic linkage of AMR genes, e.g., co-localization on the same plasmid. However, a wide confidence interval (CIs) was observed and likely due to a small sample size or variability of the study group. The significant association between AMR phenotype and genotype was observed. Positive associations were identified between phenotypic resistance to CIP-CTX-M-14, STR-*mcr-1*/ CTX-M-55, SUL-*mcr-2*/TEM-1/CTX-M-55, TET-*mcr-2*/TEM-1/CTX-M-14/CTX-M-55 and TMP-*mcr-1*/*mcr-2*/CTX-M14. This could be possible due to co-localization of multiple resistance genes on the same plasmid. The strongest association was observed between tetracycline resistance and *bla*_CTX-M-55_ (OR = 31) or *mcr-2* (OR = 9.95), in agreement with previous studies^19^. The results emphasize that emergence and spread of AMR is a dynamic issue and selective pressure of resistance to various antimicrobials are linked. Therefore, regulation of antimicrobial use should be conducted using a whole-system approach, not at individual drug level.

In conclusion, the findings emphasize the role of commensal and pathogenic *E. coli* as an important reservoir of ESBL and *mcr* genes encoding resistance to the highest priority critically important antimicrobials (HP-CIAs). Horizontal transfer of the genes indicates their significance as a global health risk. The use of ampicillin could select for colistin resistance, confirming that the pandemic spread of *mcr* genes can be a result of co-selection by other antimicrobial classes. Coexistence of genes encoding resistance to multiple clinically important antimicrobials raises a particular concern of future challenges for infection treatment options in either veterinary or human medicine. Therefore, prudent and responsible use of antibiotics in food animal production should be encouraged and whole-system approach to optimize antimicrobial uses is required. Detection of ESBL production and colistin resistance at phenotypic and genotypic level should be included in national AMR surveillance program to allow epidemiological tracing of resistance trend. Further studies to characterize *E. coli* carrying different *mcr* genes and plasmid backbones of ESBL and *mcr* genes are warranted.

## Supplementary Information

Supplementary Table 1.

Supplementary Table 2.
