# Supplementary material for: Plasmid-mediated colistin resistance and ESBL production in Escherichia coli from clinically healthy and sick pigs
Source: Sci Rep. 2022 Feb 14;12:2466. doi: 10.1038/s41598-022-06415-0 (PMC8844364; doi:10.1038/s41598-022-06415-0)
Supplement: Supplementary file 1 — Supplementary Table 1. [file 41598_2022_6415_MOESM1_ESM.docx]

| PCR and primer names | | Sequence (5’ – 3’) | Amplicon size (bp) | Reference |
| --- | --- | --- | --- | --- |
| *Mcr* genes | |  |  |  |
| MCR1-IF | | CGGTCAGTCCGTTTGTTC | 309 | [[5](#_ENREF_5)] |
| MCR1-IR | | CTTGGTCGGTCTGTA |  |  |
| MCR2-IF | | TGTTGCTTGTGCCGATTGGA | 619 | [[6](#_ENREF_6)] |
| MCR2-IR | | AGATGGTATTGTTGGTTGCTG |  |  |
| MCR3-IF | | TTGGCACTGTATTTTGCATTT | 542 | [[8](#_ENREF_8)] |
| MCR3-IR | | TTAACGAAATTGGCTGGAACA |  |  |
| MCR4-IF | | ATTGGGATAGTCGCCTTTTT | 487 | [[7](#_ENREF_7)] |
| MCR4-IR | | TTACAGCCAGAATCATTATCA |  |  |
| β-lactamase and ESBL genes | |  |  |  |
|  | *bla*_CTX-M__FW | CGATGTGCAGTACCAGTAA | 585 | [[26](#_ENREF_26)] |
|  | *bla*_CTX-M__RW | AGTGACCAGAATCAGCGG |  |  |
|  | *bla*_PSE-M__FW | GCTCGTATAGGTGTTTCCGTTT | 575 | [[27](#_ENREF_27)] |
|  | *bla*_PSE-M__RW | CGATCCGCAATGTTCCATCC |  |  |
|  | *bla*_TEM__FW | GCGGAACCCCTATTT | 964 | [[64](#_ENREF_64)] |
|  | *bla*_TEM__RW | TCTAAAGTATATATGAGTAAACTTGGTCT |  |  |
|  | *bla*_SHV__FW | TTCGCCTGTGTATTATCTCCCTG | 854 | [[25](#_ENREF_25)] |
|  | *bla*_SHV__RW | TTAGCGTTGCCAGTGYTG |  |  |
|  | *bla*_CMY-1__FW | GTGGTGGATGCCAGCATCC | 854 |  |
|  | *bla*_CMY-1__RW | GGTCGAGCCGGTCTTGTTGAA |  |  |
|  | *bla*_CMY-2__FW | GCACTTAGCCACCTATACGGCAG | 856 |  |
|  | *bla*_CMY-2__RW | GCTTTTCAAGAATGCGCCAGG |  |  |
|  | Multiplex CTX-M group 1 and group 2 |  |  | [[28](#_ENREF_28)] |
|  | MultiCTXMGp1_FW | TTAGGAARTGTGCCGCTGYA^a^ | 688 |  |
|  | MultiCTXMGp1_RW | CGATATCGTTGGTGGTRCCAT^a^ |  |  |
|  | MultiCTXMGp2_FW | CGTTAACGGCACGATGAC | 404 |  |
|  | MultiCTXMGp2_RW | CGATATCGTTGGTGGTRCCAT^a^ |  |  |
|  | CTX-M group 8/25_FW | AACRCRCAGACGCTCTAC^a^ | 326 |  |
|  | CTX-M group 8/25_RW | TCGAGCCGGAASGTGTYAT^a^ |  |  |
|  | CTX-M group 9_FW | GTGACAAAGAGAGTGCAACGG | 850 | [[29](#_ENREF_29)] |
|  | CTX-M group 9_RW | ATGATTCTCGCCGCTGAAGCC |  |  |
|  | CTX-M15_SFW | CACACGTGGAATTTAGGGACT | 876 | [[30](#_ENREF_30)] |
|  | CTX-M15_SRW | GCCGTCTAAGGCGATAAACA |  |  |

**Supplementary Table 1.** Primers used in this study.

^a^ Y = T or C; R = A or G; S = G or C; D=A, G, or T
