# Supplementary material for: Plasmid-mediated colistin resistance and ESBL production in Escherichia coli from clinically healthy and sick pigs
Source: Sci Rep. 2022 Feb 14;12:2466. doi: 10.1038/s41598-022-06415-0 (PMC8844364; doi:10.1038/s41598-022-06415-0)
Supplement: Supplementary file 2 — Supplementary Table 2. [file 41598_2022_6415_MOESM2_ESM.docx]

**Supplementary Table 2.** Antimicrobial resistance patterns of *E. coli* from healthy pigs (n=354) and clinically sick pigs (n=100) in Thailand from 2007-2018

| Resistance pattern | No. of isolates (%) | |
| --- | --- | --- |
|  | **Healthy pigs**  **(N=354)** | **Sick pigs**  **(N=100)** |
| Susceptible to all | 5 (1.4) | 0 |
| CIP | 1 (0.3) | 0 |
| CIP-GEN | 2 (0.6) | 0 |
| CIP-TET | 0 | 1 (1) |
| TET-TMP | 2 (0.6) | 0 |
| AMP-CHC-TET | 8 (2.3) | 1 (1) |
| AMP-CIP-STR | 1 (0.3) | 0 |
| AMP-COL-TET | 1 (0.3) | 0 |
| AMP-STR-TET | 2 (0.6) | 0 |
| AMP-SUL-TET | 2 (0.6) | 0 |
| CHC-TET-TMP | 11 (3.1) | 0 |
| CIP-SUL-TET | 0 | 1 (1) |
| SUL-TET-TMP | 1 (0.3) | 0 |
| AMP-CHC-CIP-TET | 2 (0.6) | 0 |
| AMP-CHC-SUL-TET | 1 (0.3) | 0 |
| AMP-CHC-TET-TMP | 6 (1.7) | 0 |
| AMP-GEN-STR-TET | 2 (0.6) | 0 |
| AMP-STR-TET-TMP | 1 (0.3) | 0 |
| AMP-STR-SUL-TET | 17 (4.8) | 0 |
| AMP-SUL-TET-TMP | 4 (1.1) | 1 (1) |
| CHC-CIP-TET-TMP | 1 (0.3) | 0 |
| CHC-SUL-TET-TMP | 1 (0.3) | 0 |
| CHC-STR-SUL-TET | 1 (0.3) | 0 |
| CHC-STR-TET-TMP | 3 (0.8) | 0 |
| CIP-COL-GEN-TET | 0 | 1 (1) |
| STR-SUL-TET-TMP | 2 (0.6) | 0 |
| AMP-CHC-COL-STR-TET | 1 (0.3) | 0 |
| AMP-CHC-SUL-TET-TMP | 9 (2.5) | 0 |
| AMP-CHC-STR-TET-TMP | 1(0.3) | 0 |
| AMP-CHC-STR-SUL-TET | 1 (0.3) | 0 |
| AMP-COL-STR-SUL-TMP | 2 (0.6) | 0 |
| AMP-COL-SUL-TET-TMP | 1 (0.3) | 0 |
| AMP-GEN-SUL-TET-TMP | 1 (0.3) | 0 |
| AMP-GEN-STR-SUL-TET | 1 (0.3) | 1 (1) |
| AMP-STR-SUL-TET-TMP | 21 (5.9) | 0 |
| CHC-CIP-COL-SUL-TET | 1 (0.3) | 0 |
| CHC-COL-STR-TET-TMP | 4 (1.1) | 0 |
| AMP-CHC-COL-STR-SUL-TET | 1 (0.3) | 0 |
| AMP-CHC-COL-SUL-TET-TMP | 2 (0.6) | 0 |
| AMP-CHC-STR-SUL-TET-TMP | 44 (12.4) | 0 |
| AMP-CHC-GEN-STR-TET-TMP | 3 (0.8) | 0 |
| AMP-CHC-GEN-SUL-TET-TMP | 3 (0.8) | 0 |
| AMP-CHC-CIP-GEN-STR-TET | 0 | 1 (1) |
| AMP-CIP-COL-SUL-TET-TMP | 1 (0.3) | 1 (1) |
| AMP-CIP-STR-SUL-TET-TMP | 1 (0.3) | 0 |
| AMP-COL-STR-SUL-TET-TMP | 1 (0.3) | 0 |
| AMP-COL-GEN-SUL-TET-TMP | 1 (0.3) | 0 |

| Resistance pattern | No. of isolates (%) | |
| --- | --- | --- |
|  | **Healthy pigs**  **(N=354)** | **Sick pigs**  **(N=100)** |
| AMP-GEN-STR-SUL-TET-TMP | 3 (0.8) | 1 (1) |
| AMP-CHC-CIP-STR-SUL-TET-TMP | 5 (1.4) | 0 |
| AMP-CHC-COL-STR-SUL-TET-TMP | 2 (0.6) | 0 |
| AMP-CIP-GEN-STR-SUL-TET-TMP | 1 (0.3) | 0 |
| AMP-COL-GEN-STR-SUL-TET-TMP | 3 (0.8) | 0 |
| AMP-COL-STR-TET-CAZ-CTX-CPD | 1 (0.3) | 0 |
| AMP-COL-GEN-STR-SUL-TET-TMP | 4 (1.1) | 0 |
| AMP-CHC-CIP-GEN-SUL-TET-TMP | 1 (0.3) | 0 |
| AMP-CIP-COL-GEN-STR-TET-TMP | 1 (0.3) | 0 |
| AMP-CHC-COL-STR-SUL-TMP-TET | 0 | 1 (1) |
| AMP-CHC-GEN-STR-SUL-TET-TMP | 24 (6.8) | 0 |
| AMP-CHC-CIP-COL-GEN-SUL-TET | 1 (0.3) | 1 (1) |
| CHC-CIP-COL-GEN-STR-SUL-TET | 1 (0.3) | 0 |
| AMP-CHC-CIP-COL-SUL-TET-TMP | 5 (1.4) | 0 |
| AMP-CHC-CIP-COL-GEN-TET-TMP | 1 (0.3) | 0 |
| AMP-CHC-CIP-GEN-SUL-TET-TMP | 0 | 1 (1) |
| AMP-CIP-COLGEN-SUL-TET-TMP | 0 | 1(1) |
| AMP-CHC-COL-GEN-STR-SUL-TET-TMP | 4 (1.1) | 1 (1) |
| AMP-CHC-CIP-COL-GEN-SUL-TET-TMP | 6 (1.7) | 1 (1) |
| AMP-CHC-CIP-COL-GEN-STR-TET-TMP | 0 | 3 (3) |
| AMP-CHC-CIP-COL-STR-SUL-TET-TMP | 13 (3.7) | 4 (4) |
| AMP-CHC-CIP-STR-SUL-TET-TMP-CPD | 1 (0.3) | 0 |
| AMP-CHC-CIP-GEN-STR-SUL-TET-TMP | 5 (1.4) | 7 (7) |
| AMP-CHC-CIP-COL-GEN-TET-CTX-CPD | 0 | 1 (1) |
| AMP-CHC-STR-SUL-TET-TMP-CTX-CPD | 0 | 1 (1) |
| AMP-CHC-GEN-SUL-TET-TMP-CTX-CPD | 1 (0.3) | 0 |
| AMP-CHC-CIP-COL-GEN-STR-TET-TMP | 2 (0.6) | 0 |
| AMP-COL-GEN-SUL-TET-CAZ-CTX-CPD | 1 (0.3) | 0 |
| AMP-CHC-CIP-COL-GEN-STR-SUL-TET-TMP | 0 | 16 (16) |
| AMP-CHC-COL-GEN-STR-TMP-CAZ-CTX-CPD | 1 (0.3) | 0 |
| AMP-CHC-COL-GEN-STR-SUL-TET-CTX-CPD | 1 (0.3) | 0 |
| AMP-CHC-COL-STR-SUL-TET-TMP-CTX-CPD | 0 | 1 (1) |
| AMP-CHC-GEN-STR-SUL-TET-TMP-CTX-CPD | 4 (1.1) | 1 (1) |
| AMP-CHC-GEN-SUL-TET-TMP-CAZ-CTX-CPD | 0 | 1 (1) |
| AMP-CHC-CIP-COL-GEN-SUL-TET-TMP-CAZ | 1 (0.3) | 0 |
| AMP-CHC-CIP-COL-GEN-STR-SUL-TET-TMP | 19 (5.4) | 2 (2) |
| AMP-CHC-CIP-COL-GEN-STR-SUL-TET-CTX | 1 (0.3) | 0 |
| AMP-CHC-CIP-COL-GEN-SUL-TET-CTX-CPD | 0 | 1 (1) |
| AMP-CHC-CIP-GEN-SUL-TET-TMP-CTX-CPD | 0 | 1 (1) |
| AMP-CHC-STR-SUL-TET-TMP-CAZ-CTX-CPD | 0 | 1 (1) |
| AMP-CIP-GEN-STR-SUL-TET-TMP-CTX-CPD | 2 (0.6) | 0 |
| AMP-CHC-CIP-COL-GEN-STR-SUL-TET-CTX-CPD | 0 | 3 (3) |
| AMP-CHC-COL-GEN-STR-SUL-TET-TMP-CTX-CPD | 3 (0.8) | 0 |
| AMP-CHC-COL-GEN-SUL-TET-TMP-CAZ-CTX-CPD | 0 | 1 (1) |
| AMP-CIP-COL-GEN-STR-TET-TMP-CAZ-CTX-CPD | 1 (0.3) | 0 |
| AMP-CIP-COL-STR-SUL-TET-TMP-CAZ-CTX-CPD | 0 | 1 (1) |
| AMP-CHC-CIP-COL-GEN-SUL-TET-TMP-CTX-CPD | 2 (0.6) | 1 (1) |
| AMP-CHC-GEN-STR-SUL-TET-TMP-CAZ-CTX-CPD | 1 (0.3) | 0 |
| AMP-CHC-CIP-COL-GEN-TET-TMP-CAZ-CTX-CPD | 1 (0.3) | 0 |
| AMP-CHC-CIP-GEN-STR-SUL-TMP-TMP-CTX-CPD | 1 (0.3) | 3 (3) |
| AMP-CHC-CIP-COL-GEN-STR-TMP-CAZ-CTX-CPD | 1 (0.3) | 0 |
| AMP-CHC-CIP-COL-GEN-STR-TET-TMP-CTX-CPD | 4 (1.1) | 0 |
| AMP-CHC-CIP-COL-GEN-STR-SUL-CAZ-CTX-CPD | 1 (0.3) | 0 |
| AMP-CHC-CIP-COL-GEN-STR-SUL-TET-TMP-CAZ | 2 (0.6) | 0 |
| AMP-CHC-CIP-COL-GEN-STR-SUL-TET-CTX-CPD | 6 (1.7) | 0 |

| Resistance pattern | No. of isolates (%) | |
| --- | --- | --- |
|  | **Healthy pigs**  **(N=354)** | **Sick pigs**  **(N=100)** |
| AMP-CHC-CIP-COL-GEN-STR-SUL-TET-TMP-CPD | 1 (0.3) | 0 |
| AMP-CHC-CIP-COL-GEN-SUL-TET-CAZ-CTX-CPD | 0 | 1 (1) |
| AMP-CHC-CIP-COL-STR-SUL-TET-CAZ-CTX-CPD | 0 | 1 (1) |
| AMP-CHC-GEN-STR-SUL-TET-TMP-CAZ-CTX-CPD | 0 | 1 (1) |
| AMP-CHC-CIP-COL-GEN-SUL-TET-TMP-CAZ-CTX-CPD | 6 (1.7) | 0 |
| AMP-CHC-COL-GEN-STR-SUL-TET-TMP-CAZ-CTX-CPD | 1 (0.3) | 0 |
| AMP-CHC-CIP-GEN-STR-SUL-TET-TMP-CAZ-CTX-CPD | 1 (0.3) | 3 (3) |
| AMP-CHC-CIP-COL-GEN-SUL-TET-TMP-CAZ-CTX-CPD | 4 (1.1) | 2 (2) |
| AMP-CHC-CIP-COL-GEN-STR-TET-TMP-CAZ-CTX-CPD | 7 (2) | 1 (1) |
| AMP-CHC-CIP-COL-GEN-STR-SUL-TET-CAZ-CTX-CPD | 1 (0.3) | 4 (4) |
| AMP-CHC-CIP-COL-GEN-STR-SUL-TET-TMP-CTX-CPD | 10 (2.8) | 3 (3) |
| AMP-CHC-CIP-COL-STR-SUL-TET-TMP-CAZ-CTX-CPD | 0 | 1 (1) |
| AMP-CHC-CIP-COL-GEN-STR-SUL-TET-TMP-CAZ-CTX-CPD | 8 (2.3) | 19 (19) |

AMP, ampicillin; CAZ, ceftazidime; CIP, ciprofloxacin; CHC, chloramphenicol; COL, colistin; CPD, cefpodoxime; CTX, cefotaxime; GEN, gentamicin; STP, streptomycin; SUL, sulfamethoxazole; TET, tetracycline; TMP, trimethoprim
